# The ventromedial prefrontal cortex in response to threat omission is associated with subsequent explicit safety memory

**DOI:** 10.1038/s41598-024-57432-0

**Published:** 2024-03-28

**Authors:** Julian Wiemer, Franziska Leimeister, Matthias Gamer, Paul Pauli

**Affiliations:** Institute of Psychology (Biological Psychology, Clinical Psychology, and Psychotherapy), University of Würzburg, Würzburg, Germany

**Keywords:** Human behaviour, Fear conditioning, Hippocampus, Long-term memory, Cortex, Prefrontal cortex

## Abstract

In order to memorize and discriminate threatening and safe stimuli, the processing of the actual absence of threat seems crucial. Here, we measured brain activity with fMRI in response to both threat conditioned stimuli and their outcomes by combining threat learning with a subsequent memory paradigm. Participants (N = 38) repeatedly saw a variety of faces, half of which (CS+) were associated with an aversive unconditioned stimulus (US) and half of which were not (CS-). When an association was later remembered, the hippocampus had been more active (than when forgotten). However, the ventromedial prefrontal cortex predicted subsequent memory specifically during safe associations (CS- and US omission responses) and the left dorsolateral prefrontal cortex during outcomes in general (US and US omissions). In exploratory analyses of the theoretically important US omission, we found extended involvement of the medial prefrontal cortex and an enhanced functional connectivity to visual and somatosensory cortices, suggesting a possible function in sustaining sensory information for an integration with semantic memory. Activity in visual and somatosensory cortices together with the inferior frontal gyrus also predicted memory performance one week after learning. The findings imply the importance of a close interplay between prefrontal and sensory areas during the processing of safe outcomes—or ‘nothing’—to establish declarative safety memory.

## Introduction

Basically, a certain amount of fear and anxiety is important for guiding everyday decisions and behavior. However, such emotional responses can also lead to unnecessary personal distress when they are exaggerated or when the impact of anxiety on our thoughts and behavior interrupts daily functioning. Some feared situations are typically not associated with the dramatic consequences that might have been anxiously anticipated, such as a flight on a plane or meeting new people. It is therefore essential for humans and other animals to own the cognitive and emotional abilities to differentiate between threatening and safe stimuli. This involves, amongst others, attention, a representation of stimulus-outcome associations, adequate working memory capacity, cognitive flexibility, inhibitory processes and integrative communication between the central and autonomic nervous system^1–5^. Understanding these mechanisms and their role in the acquisition and extinction of fear can help to gain further knowledge about how humans implement this fundamental discrimination between threat and safety, what drives inter-individual differences in this capacity and what can be done to sustainably improve it.

The combined influence of a genetic predisposition, environmental factors and associative learning mechanisms can result in elevated levels of trait anxiety or even anxiety disorders^6,7^. In some individuals anxiety persists, even after exposure therapy, which involves the repeated approach towards fear provoking stimuli and is an effective treatment for anxiety disorders in general^8,9^. Apparently, not always does this lead to long term safety memory and reduced expectancy of threat. In recent years, there has been a considerable increase of interest in gaining a deeper understanding of the underlying components of effective treatments of anxiety disorders and how to optimize therapeutic procedures^10,11^. Particularly, it has been suggested that cognitive processes such as expectancy violation play a crucial role in building a new safety memory^10,12,13^. That is, if a patient fears a stimulus and during exposure their attention is drawn to the fact that they expect something harmful to happen—but this actually does not happen—fear responses would be reduced more effectively. Even more so, the omission of threat might also induce a positive and rewarding feeling of relief^14^. Taking this theory to practice, a recent study involving 605 patients revealed that treatment outcome was predicted by the amount of change in threat expectancy due to exposure^15^.

These findings suggest that optimal threat and safety learning rely on effective processing of the actual outcomes of feared cues. Ultimately, the postulated expectancy violation and expectancy change should occur right at the moment when an outcome is expected. Myriads of studies have focused on conditioned and anticipatory responses to threat cues, since they function as a model for learned fear responses. Typically, a conditioned stimulus (CS+) signals an upcoming aversive unconditioned stimulus (US). Thus, the CS+ is paired with the US. Following several learning trials, the CS+ triggers defensive responses which should not occur towards a second, safe stimulus (CS-) that has never been paired with the US. These investigations have broadened our understanding of fear learning significantly. For example, meta-analyses have summarized abnormalities in anxious individuals, such as deficient fear inhibition towards the CS- or an extinguished CS+ (i.e., a CS+ that is no longer associated with a US), or stimuli similar to the CS+ ^16–18^.

While this paradigm serves as a tool to investigate the acquisition and extinction of fear, it is also crucial to understand the neural mechanisms behind it because they might help generating theories about the involved psychological constructs and may deliver starting points for novel interventions. Brain imaging studies identified a number of brain regions implicated in differential conditioning forming an ‘autonomic-interoceptive network’, most robustly consisting of the anterior insula, inferior frontal gyrus and anterior cingulate cortex^19^. Safety learning as implemented as the difference between an extinguished and an unextinguished CS+ was found to be associated with activity in the ventromedial prefrontal cortex (vmPFC), the dorsolateral prefrontal cortex (dlPFC) and the hippocampus^20^. It has been proposed that the anterior insula helps generate awareness of interoceptive signals and a motivational state to initiate behavioral responses further carried on by the anterior cingulate cortex, which has evolved as a motor control region^21^. Accordingly, in threat conditioning the network might enable the awareness of threat related autonomic arousal and the initiation of defensive responses. The vmPFC is functionally and structurally connected with inhibitory areas in the amygdala and rodent and human models alike propose the reduction of fear via this circuit^22^. The hippocampus is most likely essential for the contextual modulation of fear extinction^23,24^, while the dlPFC might reflect the voluntary emotion regulation via response inhibition and executive control^25^.

However, much less is known so far about the role of outcome processing. Nonetheless, according to the above-mentioned theorization, this should be important because the outcome constitutes the moment when expectancies are violated. The US is often an aversive electro-tactile stimulus and as such provokes activity in the amygdala, the insula, the somatosensory and cingulate cortices^26^. The omission of the US has been found to evoke activity in the dlPFC, the anterior cingulate cortex (ACC) and the posterior parietal cortex^27^. Another study found, amongst others, activity in the cerebellum, the insula, the inferior frontal cortex, the superior frontal medial cortex and the ACC^28^. These results refer, however, to the difference between an expected and an unexpected US omission following a still threatening CS+. That is, it may reflect more of a surprise response that generates threat learning than the actual processing of a safe outcome. Thus, we designed a brain imaging experiment in order to examine both the processing of threatening and safe outcomes and their association with threat and safety memory.

Our experiment is an application of the subsequent memory paradigm, in which an individual studies a series of items of which some will be remembered, and some will be forgotten at a later point in time. By comparing brain responses to remembered items with responses to forgotten items during learning, one can identify neural activity that is associated with successful memory encoding. In previous experiments^29,30^, we found an enhanced late positive potential (LPP) during CS+ and CS- processing, as well as larger P300 amplitudes to the US (as the CS+ offset) and the omission of the US (as the CS- offset). This supports the assumption that attentional allocation to outcomes may be crucial for explicit threat and safety memory as this component is closely related to frontal attention mechanisms^31^. The LPP as well is discussed as an indicator of motivated attention generated by connectivity between prefrontal and visual areas^32^. However, since the spatial resolution of EEG is limited, fMRI applications seem promising to further elucidate the underlying neural processes. As meta-analytic findings suggest, investigations of subsequent memory effects in non-fear related memory (as opposed to fear-related associative memory in the present study) have found memory-related activity in the inferior frontal cortex, the hippocampal formation, the fusiform cortex, the premotor area and the posterior parietal cortex. The left hippocampal region seemed to be specifically relevant for pictorial associative memory^33^.

Based on what we know so far about brain activity associated with subsequent memory and threat learning, we focused on three regions of interest in the current study: the hippocampus, the vmPFC and the dlPFC. Although its exact function in memory processes is not completely understood, it has been suggested that the hippocampus integrates relational information and by this constructs complex episodic memory traces^34,35^. During retrieval, it reinstates relational sensory and spatial information in the association cortex^36^. Following these insights, the hippocampus should be active when the relation between a CS and an outcome (US or no US) is constructed. Thus, it should play a general role in declarative memory, regardless of threat or safety. The vmPFC is related to value-based decision making, emotion regulation, latent structure learning and social cognition while the present work initially focusses on the more posterior subregion that overlaps with value and emotion processing^37,38^. Some studies suggest that the vmPFC might play a general role in the emotional modulation of memory, which is not limited to safety learning but includes threat learning as well^35,39^. For instance, patients with lesions in the vmPFC do not acquire conditioned skin conductance responses to a CS+ ^39^. However, this structure has also repeatedly been found to be involved in fear extinction, emotion regulation and positive emotional value^20,40–42^. In order to further elucidate this region’s role in safety learning, we chose a region of interest in the present analysis that has been found to be associated with extinction recall in a meta analysis^20^. It should be noted that this region is in part located in the subgenual ACC (sgACC) and somewhat more posterior than other vmPFC activations in threat and safety learning. Finally, the dlPFC has been linked to higher cognitive functions such as attentional control and working memory^43^. Related to the present work, it was found to predict contingency awareness in fear conditioning, biases in associative fear learning and more generally cognitive control and executive functions, which should be relevant for intentional, goal oriented encoding of threat and safety memory^5,44,45^. This is particularly interesting for threat learning, because dlPFC function has also been found to be affected in anxious individuals^46,47^.

Taken together, in the present experiment, we measured brain activity in healthy participants while they were intentionally learning associations between a variety of faces (as CS+ or CS-) and aversive electrical stimulations (as US following CS+) or its omission (following CS-). The timing of CS onsets and outcomes was jittered and balanced across conditions in a way that allowed us to differentiate brain responses to these events^48,49^. Our primary research questions were (1) if the hippocampus, the dlPFC and the vmPFC were associated with episodic threat and safety memory, (2) the vmPFC plays a special role in safety memory and (3) which brain activity in response to US omissions may be particularly relevant for safety learning. More specifically, due to its general role in episodic memory, we expected the hippocampus to be more active for remembered associations regardless of threat or safety^33,34^. Likewise, the dlPFC and its functional role in cognitive control and contingency monitoring should be relevant for both threat and safety learning^5,45^. The posterior vmPFC was expected to show stronger subsequent memory effects for safety than threat associations due to its role in positive emotional value and extinction recall^20,42^. While having no strong predictions about the relative impact of the onset and offset of CS+ and CS-, we expected both to reveal subsequent memory effects and specifically examined the role of US omission in exploratory analyses. To this end, we carried out exploratory whole brain analyses to capture other relevant brain regions and exploratory functional connectivity analyses based on subsequent memory effects in response to the US omission, since this event should be particularly important for safety learning^10,12,15^. Finally, we assessed associative memory one week later and examined the relationship between inter-individual differences in brain activity and memory performance in an exploratory correlational analysis.

## Method

### Participants

The current study was approved by the ethics committee of the Department of Psychology at the University of Würzburg and complied with the Declarations of Helsinki. Participants were recruited via advertisements on local boards and websites such as the University’s online registration system for psychological studies. In total, 43 participants took part in this within-subjects designed study. Three participants were excluded due to an insufficient minimum number of trials in a condition (< 12) and two participants were excluded due to a negative overall memory performance (i.e., negative d’ values due to more wrong than right answers to the question whether a CS had been presented with a US or not). The cut-off criterion for number of trials was defined post-hoc, after the distribution of memory performance was known. This led to a final sample of 38 participants (25 female; age *M* = 24.13 years, *SD* = 6.33 years). The sample size was based on our previous experiments and the according power analyses^29,30^. These power analyses suggested a required sample size of N = 34 for a statistical power of 0.80 to detect a medium sized effect of *d* = 0.5 with a two-tailed dependent *t*-test^50^. Participants had normal or corrected-to-normal vision, and by self-report, had not suffered from any psychiatric or neurological disease, nor did they take any psychoactive medication. Average state (*M* = 36.39, *SD* = 6.72) and trait anxiety (*M* = 36.66, *SD* = 9.46) according to the State Trait Anxiety Inventory (STAI)^51^, a widely used anxiety questionnaire with excellent reliability^33^, were within a normative range^52^.

### Procedure

After providing written informed consent, participants completed questionnaires on demographic data and anxiety (STAI). Afterwards, electrical stimulation electrodes were applied to the right leg and stimulation intensity was adjusted to the individual pain threshold. Then, participants engaged in the learning task in the scanner and immediately afterwards were asked to retrieve the learned associations outside the scanner. Finally, one week after learning, there was a second, internet-based, retrieval phase.

**Figure 1:**
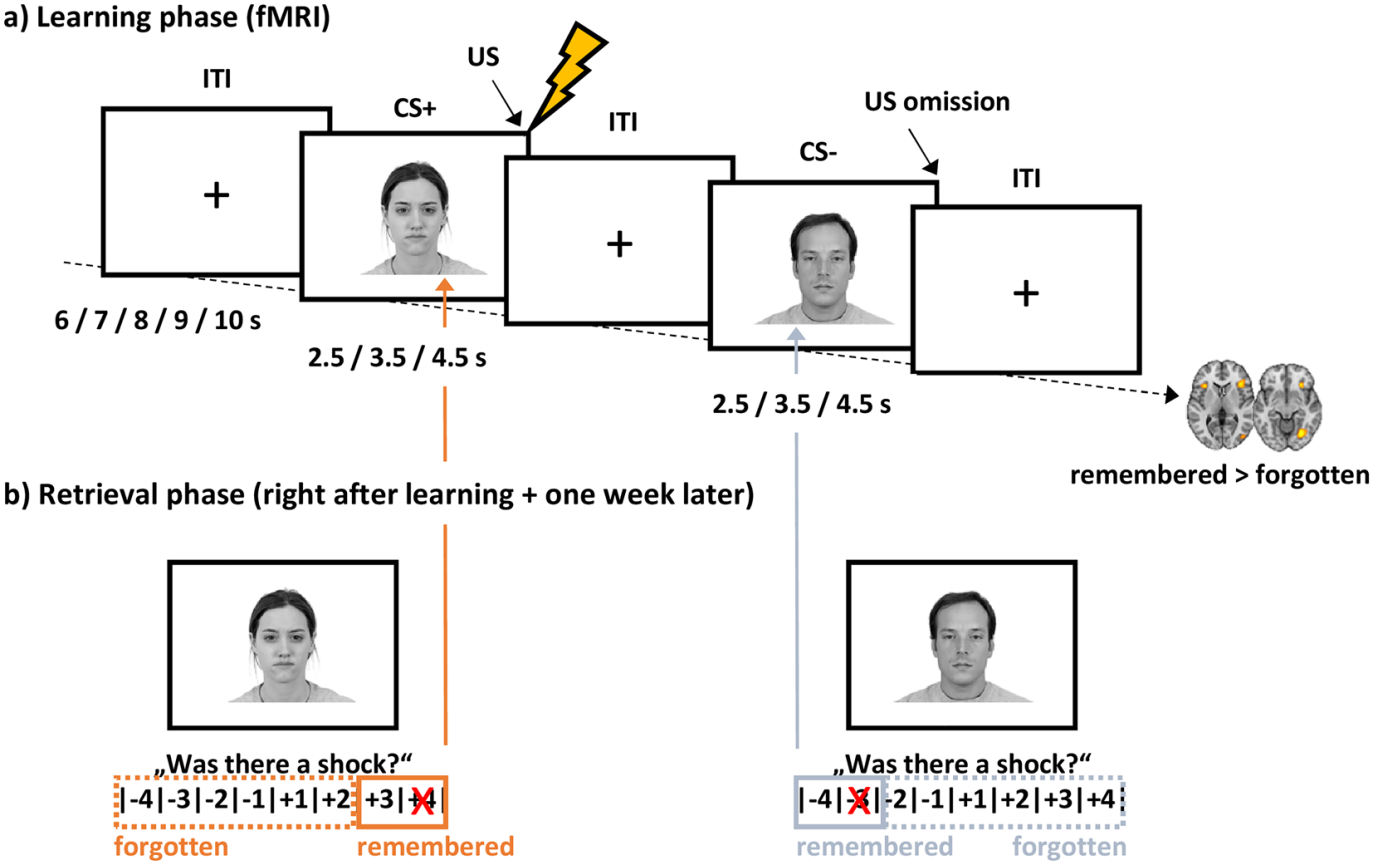
Subsequent memory design. (**a**) The learning phase took place in the scanner. Participants were instructed to memorize associations between CS and outcomes. CS durations were jittered in order to dissociate brain responses to CS onsets and outcomes. Across the experiment, each face/outcome combination was presented three times. (**b**) The retrieval phase took place outside the scanner. Participants were asked to indicate if a US had been associated with a face or not and how confident they were. Only high confident hits were assigned to the remembered category, remaining stimuli to the forgotten category. This allowed for the investigation of subsequent memory effects by comparing later remembered and forgotten trials during the learning phase. ITI = inter-trial-interval. Permission for image usage granted by the Center for Decision Research, University of Chicago.

fMRI data were continuously acquired during the learning phase that consisted of three blocks. In every block, each of 84 different faces was presented once with its associated outcome. Thus, every combination of face and outcome was presented three times. Every face was presented for three different durations (2.5, 3.5, and 4.5 s) with random assignment to the three blocks in order to temporally dissociate onsets and offsets of images. The inter-trial-interval varied between 6 and 10 s in one-second steps (see Fig. 1). Fifty percent of the images were immediately followed by an electrical stimulus (42 CS+, 42 CS-). The order of stimuli was randomized with a maximum of four consecutive trials of the same type. Between blocks, participants had the opportunity to take a short rest and continued whenever they were ready. Participants were specifically instructed to memorize which faces were associated with a US and which were not. They were also informed about the up-coming retrieval test.

In the retrieval phase that was accomplished outside the scanner, participants were presented with each of the 84 pictures again, for at least two seconds, and asked to indicate if there had been a US associated with a given picture. Simultaneously, they rated their confidence on a scale from − 4 (very certain no US) to + 4 (very certain US), leaving out zero (i.e., forced choice to avoid a central tendency in uncertain trials). Valence (from 0 [very unpleasant] to 100 [very pleasant]) and arousal ratings (from 0 [calm] to 100 [very arousing]) were also collected for each picture. Seven days later, participants were sent an online link to a second follow-up memory rating.

### Stimuli

Eighty-four different faces with a neutral expression were derived from the Chicago Face Database^53^ and used as CS+ and CS-. Two sets of 42 stimuli each were hand-selected while making sure that the sets contained an equal number of male and female faces, and did not differ in age and normatively rated attractiveness. In addition, the images were converted to black and white with no differences in luminance between the stimulus sets, as indicated by a two-sided *t*-test (*M* ± *SD* = 0.82 ± 0.03 vs. *M* ± *SD* = 0.82 ± 0.03), *t*(82) = 0.20, *p* = 0.84, *d* = 0.04, 95% CI [− 0.015, 0.012]. Alternating between participants, one set served as CS+ and the other one as CS-. The images were successfully used as CS before^29,30^.

Mildly painful electrical stimuli were generated by a constant current stimulator (Digitimer DS7A; Digitimer Ltd, Welwyn Garden City, UK) and served as US. They were applied via two steel surface electrodes (9-mm diameter; GVB-geliMED, Bad Segeberg, Germany) to the inner side of the right calf. The voltage was set at 400 V with alternating polarity between pulses. A US consisted of 10 pulses of 2 ms interleaved with 2 ms breaks of no stimulation, resulting in a 40 ms lasting electro-tactile stimulation. The intensity of the current was adjusted to each individual’s pain threshold, which was determined prior to the experimental procedure, while participants were lying on the scanner bed. To determine pain thresholds, different currents were applied in two ascending and two descending sequences with steps of 0.2 mA, while participants rated the painfulness of the stimuli on a scale from 0 (= no sensation) to 10 (= maximal painful sensation). Participants were instructed that a rating of 4 indicated the beginning of painful sensation. This procedure resulted in a mean current of 1.87 mA (*SD* = 0.56 mA).

### Behavioral data analysis

Memory and emotion ratings were analyzed with repeated measures ANOVAs more specifically described in the results section. Significant effects were followed up by ANOVAs with lower complexity and ultimately paired *t*-tests, which were two-sided, if not otherwise specified. Mean values are reported alongside (±) standard deviations. Memory ratings right after learning were analyzed with a paired *t*-test between hits (CS+) and false alarms (CS-). In addition, d’ values were calculated as the standardized difference in z-values between hits and false alarms in the retrieval phase (see Fig. 1). This was done to analyze the basic ability to discriminate between CS+ and CS- if the tendency of a response was correct (positive or negative), regardless of confidence. The significance threshold was set at 0.05. As a memory performance measure, we calculated the means of the individual memory ratings for CS+ minus the means of the individual memory ratings for CS-, resulting in a potential range from − 8 to 8 with 8 reflecting perfect memory and confidence (see also^30^). This was also done to correlate brain activity with memory performance one week after learning (see below). After one week, memory became overall worse but a few individuals excelled in performance, which resulted in a left-skewed non-normal distribution. Therefore, we took the natural logarithm of memory performance in order to normalize the distribution, set the requirements for correlational analyses and protect against outlier driven artifacts in the fMRI analyses. A comparison of log-transformed and non-transformed analyses confirmed that random voxels outside the brain attributable to single outliers were removed by this process.

### fMRI measurement and analysis

#### Imaging parameters

Whole brain functional imaging was collected on a 3 T Siemens Skyra at the Magnetic Resonance Department of the Fraunhofer Institute for Integrated Circuits Würzburg, Germany. A gradient echo field mapping was performed to prepare for distortion correction in preprocessing. Echo-planar images (EPIs) were acquired in three sessions of 490 images and a duration of 16:20 min. each with the following parameters: repetition time = 2 s, echo time = 29 ms, flip angle = 90°, acquisition matrix = 64 × 64, 33 axial slices, voxel dimensions = 3.28 × 3.28 × 3.80 mm, 0.38 mm gap. Axial slices were aligned to the anterior and posterior commissure. The task started after the first five EPIs to allow for a stabilization of the magnetic field. After the third functional session, a high resolution structural T1-weighted image was created in a Magnetization Prepared Rapid Gradient Echo (MPRAGE) sequence with the following parameters: repetition time = 2.3 s, echo time = 2.96 ms, inversion time = 1.1 s, flip angle = 9°, acquisition matrix = 256 × 192, 240 axial slices, voxel dimensions = 1 × 1 × 1 mm.

#### Preprocessing

FMRI data were analyzed using Statistical Parametric Mapping software (SPM12; Wellcome Department of Imaging Neuroscience, London, UK). The first five volumes of each session were discarded from the analysis to allow for a stabilization of the magnetic field. Remaining functional images were realigned and unwarped using an individual voxel displacement map on the basis of individual field mapping. Then, they were slice time corrected and co-registered to the mean of individual functional images and structural scans. We then used the ArtRepair toolbox^54^ in order to correct for residual head movement that might not have been properly corrected by the previous realignment procedure. In this step, images were slightly smoothed (4 mm FWHM Gaussian kernel) and motion adjusted using six motion regressors derived from realignment parameters. Then, data with high scan-to-scan movement (> 0.5 mm) or total movement (> 3 mm) were repaired by interpolation between the nearest non-repaired scans. Finally, additional spikes in the signal intensity were removed (> 4% of a rolling mean). Such procedure was shown to improve signal quality and yield more robust results^54^. Volumes were then normalized to MNI space using tissue segmentation (voxel dimensions 2 × 2 × 2 mm) and smoothed again (8 mm FWHM Gaussian kernel).

#### First level analysis

For within subject memory analysis, the model included eight regressors of zero duration impulse responses. Four regressors were dedicated to the onsets of CS (CS+ remembered, CS+ forgotten, CS- remembered, CS- forgotten) and four regressors modeled responses to the offsets of the CS (US remembered, US forgotten, US omission remembered, US omission forgotten). Thus, in total, the model included eight regressors of interest: CS+ remembered (the onset of a CS+ that was later correctly associated with a US [memory rating ≥ 3]), CS+ forgotten (the onset of a CS+ that was later not associated with a US [memory rating < 3]), CS- remembered (the onset of a CS- that was later correctly associated with no US [memory rating ≤ -3]), CS- forgotten (the onset of a CS- that was later not associated with no US [memory rating > − 3]), US remembered (the offset of a CS+ that was later correctly associated with a US [memory rating ≥ 3]), US forgotten (the offset of a CS+ that was later not associated with a US [memory rating < 3]), US omission remembered (the offset of a CS- that was later correctly associated with no US [memory rating ≤ − 3]), US omission forgotten (the offset of a CS- that was later not associated with no US [memory rating > − 3]). No nuisance regressors were incorporated because movement correction was applied during preprocessing. The separation of remembered and forgotten associations was based on the memory rating assessed immediately after scanning. In accordance with previous subsequent memory studies, only high confidence items were classified as remembered (rating of + 3 and + 4 for CS+ and rating of − 3 and − 4 for CS-) and low confidence items were classified as forgotten (below + 3 and above − 3, resp.), because we were primarily interested in the basis of stable (safety) memory. This procedure is in accordance with standard procedures in subsequent memory paradigms^55^ and our previous experiments^29,30^. With a total of 252 trials, the average number of trials per condition were 61.67 remembered CS+, 64.34 forgotten CS+, 55.26 remembered CS- and 70.74 forgotten CS-. The calculation of variance inflation factors (VIF) indicated that regressors were widely independent (with a mean of 1.21 and a maximum of 1.24) and values were well below recommended thresholds (< 5)^56^.

In order to analyze the correlation between brain activity and inter-individual memory performance, we set up a second model with only four regressors (CS+, CS-, US, US omission). Memory status was not included in the first level model, but later introduced as a covariate on second level. To this end, a memory performance measure was calculated for each participant (see behavioral data analysis). In both models, a high-pass 128 s filter was applied to account for low-frequency drifts.

#### Psychophysiological interaction (PPI)

Since we were primarily interested in subsequent memory effects in response to the omission of the US after the CS-, we investigated functional connectivity with the medial prefrontal cortex (MPFC) as a follow-up test to the most prominent result in this regard (see Results section and Fig. 4a). The seed region was defined on an individual basis by a sphere (*r* = 6 mm) around the individual peak activation within an MPFC mask at the contrast [US omission remembered > US omission forgotten]. The MPFC mask consisted of significantly activated voxels at this contrast at the group level (see Results section; *p* < 0.001, cluster size *k* ≥ 126) within the borders of the medial frontal gyrus as defined anatomically according to the Talairach Daemon database^57^. While the time series of the seed region served as the physiological variable, the contrast [US omission remembered > US omission forgotten] was included as the psychological variable. Results were thresholded similarly as for the other whole brain analyses.

#### Second level analysis

On second-level, regions of interest (ROIs) were defined based on previous findings in subsequent memory, executive control, and fear extinction research. All ROIs were constructed as 10 mm spheres centered around peak coordinates in meta-analytic reports. For the hippocampus, we used [− 22 − 10 − 16] for the left side and [− 18 − 8 − 16] for the right side, as reported by Kim^33^ in a meta-analysis of 74 fMRI studies on subsequent memory effects. For the dlPFC, we used [− 38 6 28] for the left side and [40 26 22] for the right side, as reported by Niendam and colleagues^45^ in a meta-analysis of 193 imaging studies on cognitive control. Specifically, we used the results of a conjunction analysis comprising working memory, flexibility and inhibition. Finally, for the vmPFC, we used [− 4 34 − 6], as reported by Fullana and colleagues^20^ in a meta-analysis of fear extinction recall studies in which an extinguished CS+ was compared with an unextinguished CS+. Note, that this ROI is located at the posterior of the vmPFC, respectively in the sgACC. For each ROI and each of the eight regressors (CS+ remembered, CS+ forgotten, CS- remembered, CS- forgotten, US remembered, US forgotten, US omission remembered, US omission forgotten), we extracted the first eigenvariate of each participant as an index of brain activity in that region. Then, we tested for subsequent memory effects via repeated measures ANOVAs and *t*-tests as further specified in the results section. The significance threshold was set at 0.05.

We also examined subsequent memory effects in the whole brain by analyzing one sample t-tests of the contrasts [CS+ remembered > CS+ forgotten], [CS- remembered > CS- forgotten], [US remembered > US forgotten] and [US omission remembered > US omission forgotten]. To explore potential interaction effects, we also explored the contrasts [[CS+ remembered > CS+ forgotten] vs. [CS- remembered > CS- forgotten]] and [[US remembered > US forgotten] vs. [US omission remembered > US omission forgotten]] in both directions. For whole brain analyses, a combined threshold was set to an initial voxel-vise threshold of *p* < 0.001 and a cluster threshold of *k* ≥ 126. A Monte Carlo simulation including original voxel size, acquisition matrix and smoothing parameters was run with 10,000 repetitions to determine this threshold^58^. Anatomic labels are reported according to the automatic anatomic labelling atlas 3^59^.

## Results

### Memory and emotion ratings

#### Memory

**Figure 2:**
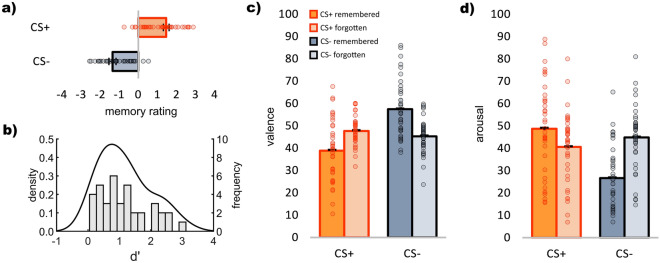
Memory and emotion ratings of CS+ and CS-. (**a**) Right after the learning phase, CS+ was more associated with the US than CS- (4 = very certain that US had been following the CS; − 4 = very certain that US had not been following the CS). (**b**) Kernel density distribution and histogram of d’ values for individual participants indicate memory performance right after learning. Two participants with negative values had been excluded. (**c**) CS+ was rated more negative when remembered than when forgotten and CS- was rated more positive when remembered than when forgotten. (**d**) Likewise, CS+ was rated more arousing when remembered and CS- less arousing when remembered. Error bars indicate standard errors of the mean.

CS+ (*M* = 1.46 ± *SD* = 1.15) was rated significantly more positive on the memory and confidence scale than CS- (*M* = − 1.35 ± *SD* = 0.95), *t*(38) = 9.69, *p* < 0.001, *d* = 1.57, 95% CI [2.22, 3.40], i.e. it was associated with the US more often or with more confidence than the CS-. Both CS+ , *p* < 0.001, and CS- significantly diverged from zero, *p* < 0.001 (see Fig. 2a). This indicates that on average, participants were better than chance in associating a CS+ with a US and a CS- with the omission of a US. On average, 49% of CS+ and 44% of CS- were classified as remembered, with the difference not being statistically significant, *p* = 0.064. d’ values ranged from 0.18 to 3.12 (*M* = 1.24 ± *SD* = 0.76; see Fig. 2b).

#### Valence

A repeated measures ANOVA with the factors *CS* (CS+ , CS-) and *memory* (remembered, forgotten) revealed a significant main effect of *CS*,* F*(1,37) = 22.74, *p* < 0.001, *η*_*p*_^*2*^ = 0.38, and a significant interaction between *CS* and *memory*, *F*(1,37) = 33.49, *p* < 0.001, *η*_*p*_^*2*^ = 0.48. Remembered CS- (*M* = 57.39 ± *SD* = 12.71) were rated as more positive than forgotten CS- (*M* = 45.22 ± *SD* = 7.53), *t*(37) = 5.26, *p* < 0.001, *d* = 0.85, 95% CI [7.48, 16.85], while remembered CS+ (*M* = 38.80 ± *SD* = 13.70) were rated as more negative than forgotten CS+ (*M* = 47.66 ± *SD* = 6.33), *t*(37) = 4.07, *p* < 0.001, *d* = 0.66, 95% CI [4.45, 13.28] (see Fig. 2c).

#### Arousal

A repeated measures ANOVA with the factors *CS* (CS+ , CS-) and *memory* (remembered, forgotten) resulted in a significant main effect of *CS*,* F*(1, 37) = 20.88, *p* < 0.001, *η*_*p*_^*2*^ = 0.36, a significant main effect of *memory*, *F*(1, 37) = 5.67, *p* = 0.023, *η*_*p*_^*2*^ = 0.13, and a significant interaction between *CS* and *memory*,* F*(1, 37) = 34.83, *p* < 0.001, *η*_*p*_^*2*^ = 0.49. Remembered CS+ (*M* = 48.72 ± *SD* = 20.77) were reported with significant higher arousal than forgotten CS+ (*M* = 40.58 ± *SD* = 15.79), *t*(37) = 2.36, *p* = 0.023, *d* = 0.38, 95% CI [1.17, 15.12], while remembered CS- (*M* = 26.62 ± *SD* = 13.95) were reported with significant less arousal than forgotten CS- (*M* = 44.81 ± *SD* = 14.33), *t*(37) = 6.87, *p* < 0.001, *d* = 1.12, 95% CI 12.83, 23.55] (see Fig. 2d).

### Regions of interest analyses

ROI analyses were conducted by separate repeated measures ANOVAs for the three ROIs (hippocampus, vmPFC, dlPFC), including the factors timing (CS onset [+ and −], CS outcome [US and US omission]), US pairing (yes [CS+ and US], no [CS- and US omission]), memory (remembered, forgotten) and (for hippocampus and dlPFC) laterality (left, right). Timing refers to whether the response was locked to the onset of a picture or the offset of a picture, regardless of whether it was CS+ or CS-. US pairing refers to whether a US occurred in a trial, regardless of whether the response was locked to the onset or offset of a picture. Finally, laterality refers to the left or right hemisphere of the brain, while for the vmPFC, due to its medial location, laterality was not considered.

#### Hippocampus

**Figure 3:**
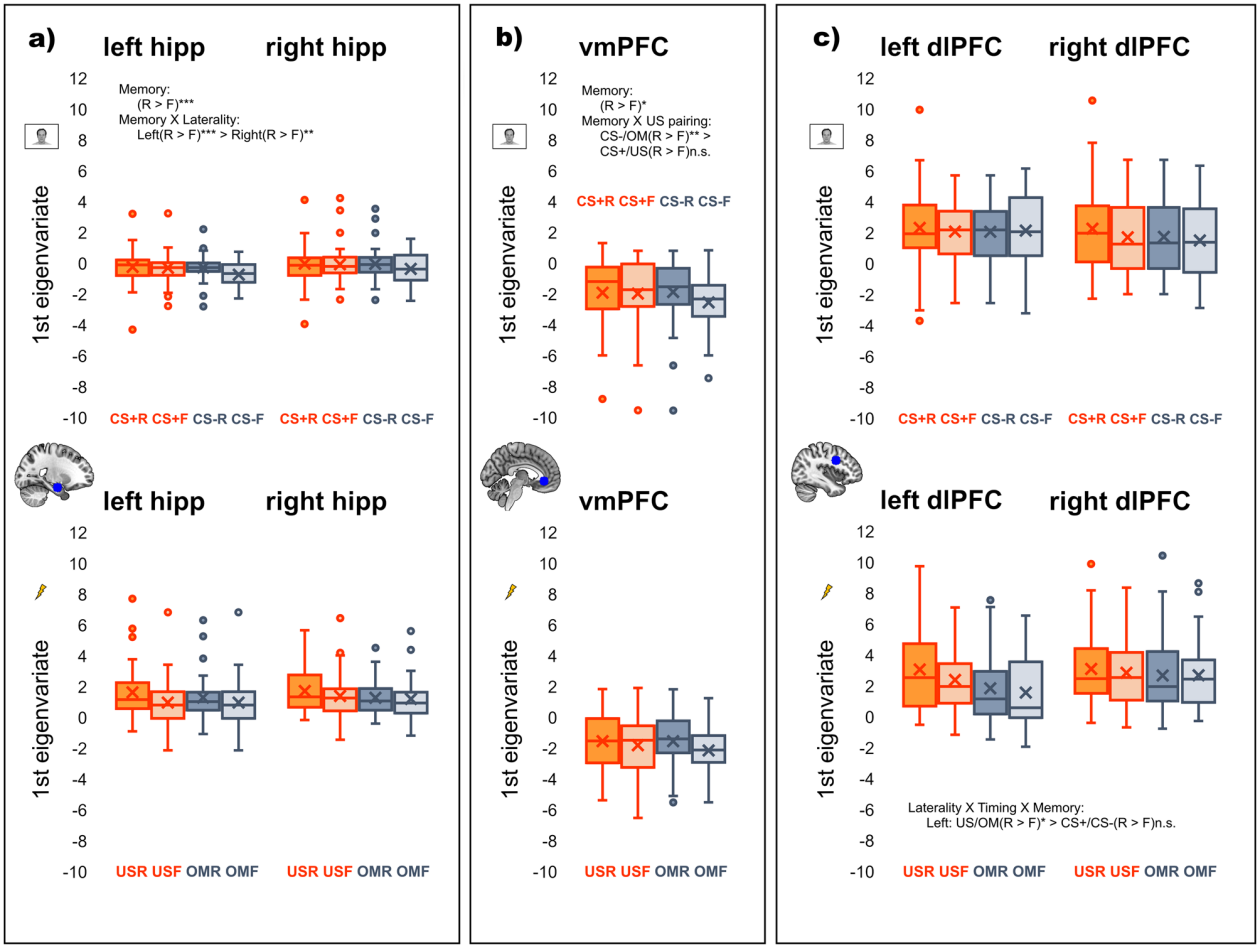
Regions of interest (ROI) results. Upper row shows CS+ and CS- responses, lower row shows US and US omission responses. Only ANOVA memory effects are described. (**a**) An overall subsequent memory effect in the hippocampus was more pronounced on the left side than on the right side. (**b**) The subsequent memory effect in the vmPFC was only present for safe trials (CS-/US omission). (**c**) The subsequent memory effect in the dlPFC was restricted to the left side and outcome responses (US/US omission). hipp = hippocampus, vmPFC = ventromedial prefrontal cortex, dlPFC = dorsolateral prefrontal cortex, R = remembered, F = forgotten, US = unconditioned stimulus, OM = omission of unconditioned stimulus.

There were significant main effects of timing, *F*(1, 37) = 69.16, *p* < 0.001, *η*_*p*_^*2*^ = 0.65, US pairing, *F*(1, 37) = 12.15, *p* = 0.001, *η*_*p*_^*2*^ = 0.25, and memory, *F*(1, 37) = 15.57, *p* < 0.001, *η*_*p*_^*2*^ = 0.30, also an interaction between laterality and memory, *F*(1, 37) = 10.77, *p* = 0.002, *η*_*p*_^*2*^ = 0.23. Other effects were non-significant, *p*s > 0.075. On average, the hippocampus was more responsive to outcomes (*M* = 1.34 ± *SD* = 1.25) than to onsets (*M* = -0.25 ± *SD* = 0.96), to paired (*M* = 0.67 ± *SD* = 1.08) than unpaired events (*M* = 0.42 ± *SD* = 0.85), and to remembered (*M* = 0.67 ± *SD* = 0.99) than to forgotten events (*M* = 0.42 ± *SD* = 0.94). Although, this subsequent memory effect was significant for both the left (remembered: *M* = 0.61 ± *SD* = 1.05, forgotten: *M* = 0.30 ± *SD* = 0.95), *t*(37) = 4.45, *p* < 0.001, *d* = 0.72, 95% CI [0.17, 0.45], and the right hippocampus (remembered: *M* = 0.74 ± *SD* = 1.03, forgotten: *M* = 0.54 ± *SD* = 1.04), *t*(37) = 3.09, *p* = 0.002, *d* = 0.50, 95% CI [0.07, 0.32], it was stronger in the left hemisphere (left: *M*_*∆*_ = 0.31 ± *SD* = 0.43, right: *M*_*∆*_ = 0.19 ± *SD* = 0.39), *t*(37) = 3.28, *p* = 0.001, *d* = 0.53, 95% CI [0.04, 0.19] (see Fig. 3a).

#### vmPFC

There was a significant main effect of memory, *F*(1, 37) = 9.37, *p* = 0.004, *η*_*p*_^*2*^ = 0.20, and a significant interaction between memory and US pairing, *F*(1, 37) = 5.08, *p* = 0.03, *η*_*p*_^*2*^ = 0.12. No other effects were significant, *p*s > 0.074. Only for unpaired events (i.e. CS- and US omission), the vmPFC was significantly more activated in response to remembered (*M* = − 1.70 ± *SD* = 1.39) than to forgotten associations (*M* = − 2.34 ± *SD* = 1.24), *t*(37) = 4.88, *p* = 0.001, *d* = 0.79, 95% CI [0.38, 0.91], but not for paired events (i.e. CS+ and US), *p* = 0.42 (see Fig. 3b).

#### dlPFC

There was a significant main effect of US pairing, *F*(1, 37) = 28.87, *p* < 0.001, *η*_*p*_^*2*^ = 0.44, and significant interactions between laterality and timing, *F*(1, 37) = 7.71, *p* = 0.009, *η*_*p*_^*2*^ = 0.17, laterality and US pairing, *F*(1, 37) = 5.48, *p* = 0.025, *η*_*p*_^*2*^ = 0.13, laterality, timing and US pairing, *F*(1, 37) = 12.24, *p* = 0.001, *η*_*p*_^*2*^ = 0.25, and laterality, timing and memory, *F*(1, 37) = 20.14, *p* < 0.001, *η*_*p*_^*2*^ = 0.35. No other effects were significant, *p*s > 0.072. The existent interactions were further resolved by two separate ANOVAs, one for the left and one for the right hemisphere. This, for the left side, revealed a significant main effect of US pairing again, *F*(1, 37) = 26.52, *p* < 0.001, *η*_*p*_^*2*^ = 0.42, a significant interaction between timing and US pairing, *F*(1, 37) = 12.38, *p* = 0.001, *η*_*p*_^*2*^ = 0.25, and a significant interaction between timing and memory, *F*(1, 37) = 4.23, *p* = 0.047, *η*_*p*_^*2*^ = 0.10. Overall, the left dlPFC was more responsive to paired (*M* = 2.47 ± *SD* = 1.99) than to non-paired events (*M* = 1.91 ± *SD* = 1.69). However, as qualified by the interaction with timing, this only held true for the US (*M* = 2.73 ± *SD* = 2.31) evoking stronger dlPFC activity than the US omission (*M* = 1.71 ± *SD* = 2.19), *t*(37) = 5.06, *p* < 0.001, *d* = 0.82, 95% CI [0.61, 1.43], but there was no difference between CS+ and CS-, *p* = 0.49. In terms of subsequent memory, only for outcome responses, remembered associations (*M* = 2.46 ± *SD* = 2.45) resulted in stronger left dlPFC activity than forgotten associations (*M* = 1.99 ± *SD* = 2.04), *t*(37) = 2.30, *p* = 0.027, *d* = 0.37, 95% CI [0.06, 0.89], but there was no such memory effect for CS onsets, *p* = 0.57. For the right dlPFC, there was no significant memory effect or interaction with memory, only main effects of timing, *F*(1, 37) = 7.29, *p* = 0.01, *η*_*p*_^*2*^ = 0.17, and US pairing, *F*(1, 37) = 15.63, *p* < 0.001, *η*_*p*_^*2*^ = 0.30. CS outcomes (*M* = 2.88 ± *SD* = 1.96) evoked in general stronger right dlPFC activity than CS onsets (*M* = 1.79 ± *SD* = 2.45), and paired events (*M* = 2.50 ± *SD* = 1.85) triggered stronger right dlPFC activity than non-paired events (*M* = 2.17 ± *SD* = 1.87; see Fig. 3c).

### Exploratory whole brain analyses

#### US omission

**Figure 4:**
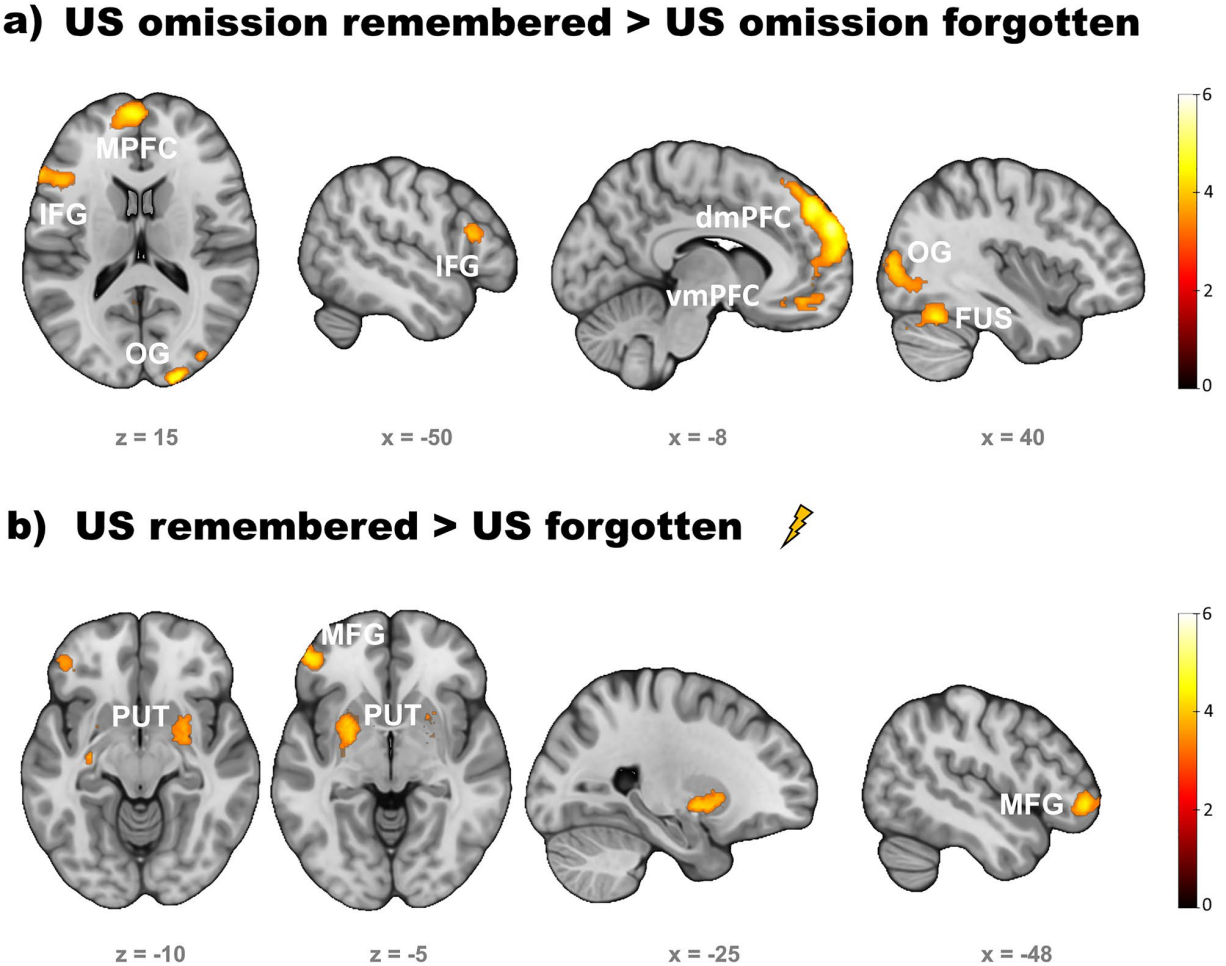
Subsequent memory effects (SMEs) in response to the US omission (**a**, after CS-) and the US (**b**, after CS+). SMEs in response to US omission following CS- included clusters in the ventromedial (vmPFC) and dorsomedial (dmPFC) prefrontal cortex, left inferior frontal gyrus (IFG), and right fusiform (FUS) and occipital gyrus (OG). US response in the left and right striatum (putamen, PUT), as well as left middle frontal gyrus (MFG) was associated with later memory. Corrected to *p* < .05 by voxel threshold of *p* < .001 and cluster extent of *k* ≥ 126.

The omission of the US following remembered as compared to forgotten CS- evoked activity in left superior and medial frontal gyrus (MPFC; including the vmPFC), left inferior frontal gyrus, right middle occipital and fusiform gyrus, right lingual gyrus, left superior temporal gyrus and posterior cingulate (see Fig. 4a and Table 1).

**Table 1:** Subsequent memory effects in outcome responses.

Region	% of	Cluster	Peak	Peak voxel		
	cluster	size	T-value	x	y	z
US omission remembered > forgotten						
Medial superior frontal gyrus left	46.58	2046	5.44	-8	58	30
Superior frontal gyrus left	33.53					
Medial orbital superior frontal gyrus left	6.55					
Cerebellum crus 1 right	64.66	348	4.99	18	-84	-28
Cerebellum right	19.83					
Cerebellum crus 2 right	15.52					
Middle occipital gyrus right	33.76	1016	4.83	24	-98	14
Fusiform gyrus right	14.86					
Superior occipital gyrus right	10.93					
Vermis	28.01	282	4.67	4	− 68	− 4
Lingual gyrus right	28.01					
Vermis	25.89					
Triangular inferior frontal Gyrus left	83.41	217	4.38	− 60	24	14
Opercular inferior frontal gyrus left	11.06					
Outside	5.53					
US remembered > forgotten						
Putamen left	44.71	624	5.14	-12	10	10
Caudate nucleus left	24.20					
Pallidum left	14.90					
Inferior frontal gyrus pars orbitalis	38.54	205				
Middle frontal gyrus	29.27					
Triangular inferior frontal gyrus	23.90		4.50	− 48	46	− 4
Outside	8.29					
Outside	45.45	132	3.97	26	2	− 10
Putamen right	35.61					
Amygdala right	14.39					

Table shows the three regions with the highest number of active voxels within each cluster, labels according to automated anatomic labelling atlas 3 (AAL3), MNI coordinates.

##### US

Separately from CS onsets we also analyzed responses to CS outcomes, i.e. US and US omission responses. Enhanced activity to the US was observed when CS+ /US associations were later remembered (vs. forgotten) in the left and right striatum, specifically in the putamen, within a cluster also involving the right amygdala. Moreover, left middle frontal gyrus was activated (see Fig. 4b and Table 1).

#### *CS+and CS- onsets*

**Figure 5:**
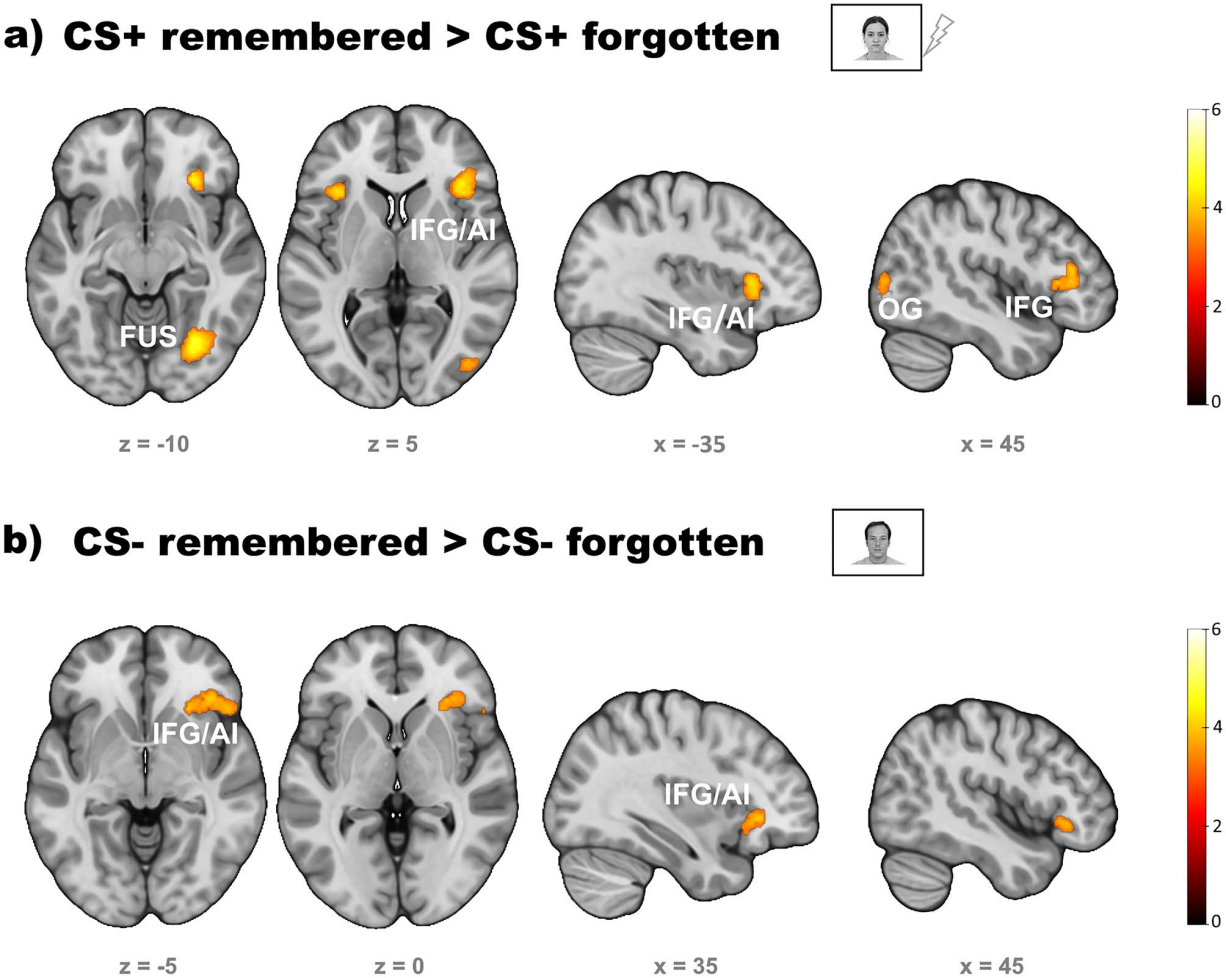
Subsequent memory effects in response to CS+ (**a**) and CS- (**b**). In both CS+ and CS- learning trials the right inferior frontal gyrus and the anterior insula were more activated the association with a US (or the omission of a US for CS-, respectively) when subsequently remembered. In CS+ additional activity was measured in visual processing areas, such as the right fusiform (FUS) and occipital gyrus (OG), the brainstem (BS) and supplementary motor area (SMA). Corrected to *p* < .05 by voxel threshold of *p* < .001 and cluster extent of *k* ≥ 126.

Whole brain analyses revealed enhanced activity in response to remembered CS+ vs. forgotten CS+ in right fusiform gyrus, right and left inferior frontal gyrus (IFG) including the anterior insula, the right middle and superior occipital gyrus, the left supplementary motor area and the brainstem. Similarly, remembered CS- also elicited stronger activity in the right inferior frontal gyrus than forgotten CS- (see Fig. 5 and Table 2).

**Table 2:** Subsequent memory effects in CS+ and CS- responses.

Region	% of	Cluster	Peak	Peak voxel		
	cluster	size	T-value	x	y	z
CS+ remembered > forgotten						
Fusiform gyrus right	64.84	401	4.95	28	− 68	− 8
Inferior occipital gyrus right	16.21					
Outside	11.47					
Triangular inferior frontal gyrus right	39.38	518	4.94	38	28	4
Insula right	28.19					
Inferior frontal gyrus pars orbitalis right	15.44					
Outside (brainstem)	100.00	106	4.60	− 12	− 32	− 34
Insula left	56.32	174	4.35	− 34	22	6
Triangular inferior frontal gyrus left	41.95					
Inferior frontal gyrus pars orbitalis left	1.72					
Middle occipital gyrus right	99.12	228	4.31	40	-80	12
Inferior occipital gyrus right	0.88					
CS-remembered > forgotten						
Inferior frontal gyrus pars orbitalis right	42.02	445	4.36	26	24	− 6
Outside	25.84					
Insula right	19.33					

Table shows the three regions with the highest number of active voxels within each cluster, labels according to automated anatomic labelling atlas 3 (AAL3), MNI coordinates.

#### Interaction contrasts

There were no significant differences between the subsequent memory effects to US and US omissions, or between CS+ and CS- onsets.

### Exploratory psychophysiological interaction (PPI) analysis

**Figure 6:**
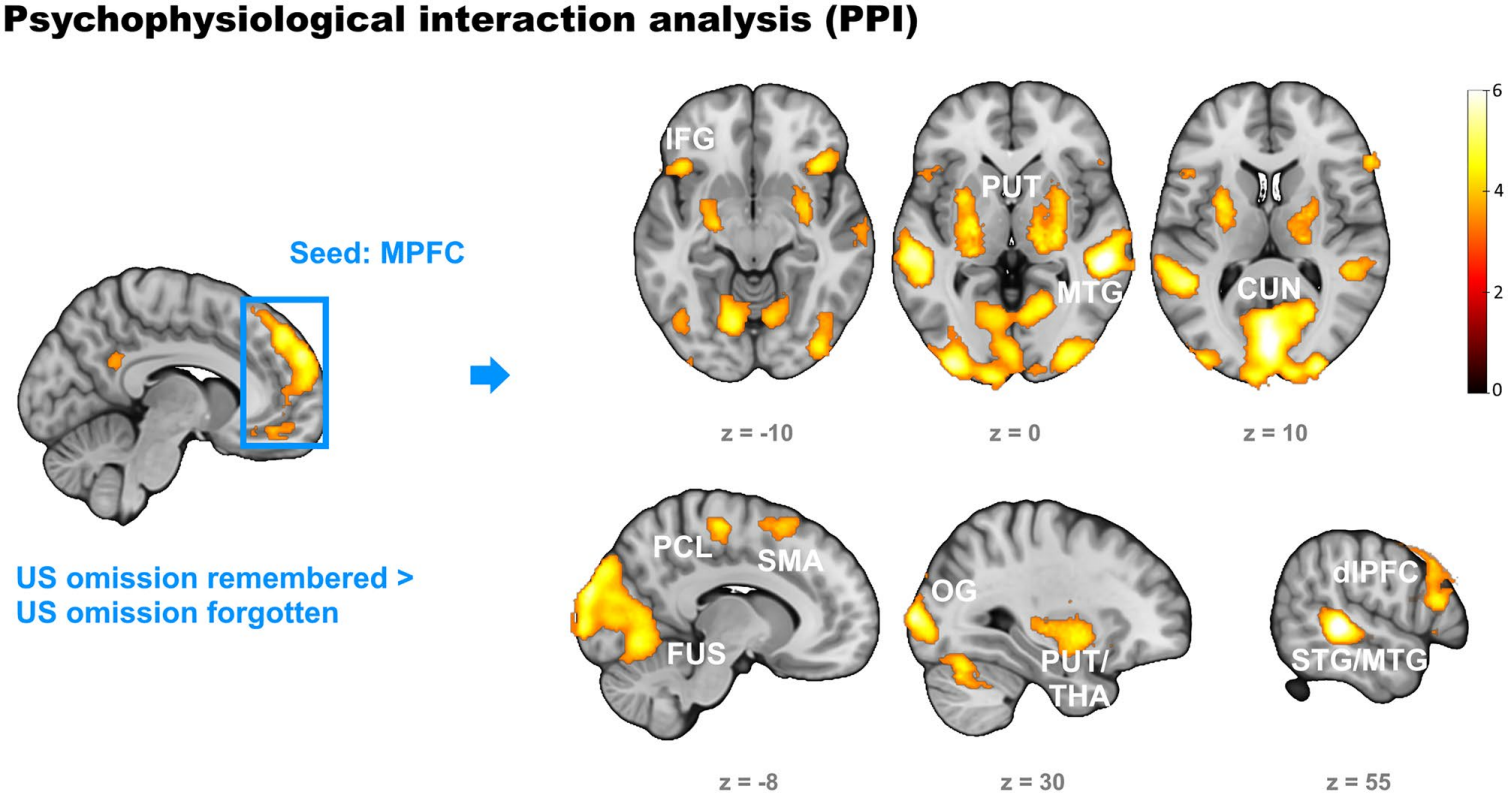
Results of the Psychophysiological interaction analysis with the MPFC significantly activated in the contrast [US omission remembered > US omission forgotten] as the seed region and the same contrast as the psychological variable. Activations on the right represent enhanced functional connectivity to the MPFC when the US omission was remembered. They comprise the inferior frontal gyrus (IFG), the bilateral putamen (PUT) and thalamus (THA), the superior and medial temporal gyrus (STG, MTG), the cuneus (CUN), the fusiform gyrus (FUS), the occipital gyrus (OG) including visual cortices, the paracentral lobule (PCL) including the primary somatosensory cortex, the supplementary motor area (SMA) and the dorsolateral prefrontal cortex (dlPFC). Corrected to *p* < .05 by voxel threshold of *p* < .001 and cluster extent of *k* ≥ 126.

Since we were primarily interested in the memory of safe outcomes, we took the subsequent memory effects of omission responses in the MPFC as a seed region (and US omission remembered > US omission forgotten as the psychological variable) to gain further insights in functionally connected brain areas. These areas turned out to be the left and right middle and superior temporal cortex, areas in the visual cortex involving the cuneus, the occipital cortex and the fusiform cortex, the right dlPFC, the left and right putamen including thalamus, hippocampus and amygdala, the left precentral cortex, the left and right supplementary motor area, the left and right paracentral lobule (as the assumed primary sensory cortex processing the US applied to the right leg) and the left and right IFG. Significant connectivity was also found between the MPFC and the predefined region of interest in the left hippocampus (see Fig. 6 and Table 3).

**Table 3:** Connectivity (PPI) with medial frontal gyrus at US omission remembered > forgotten.

Region	% of	Cluster	Peak	Peak voxel		
	cluster	size	T-value	x	y	z
Middle temporal gyrus right	50.32	1268	6.94	54	− 34	2
Superior temporal gyrus right	46.37					
Outside	3.31					
calcarine gyrus left	10.47	10,926	6.61	2	− 82	12
Calcarine gyrus right	8.96					
Cuneus left	8.43					
middle frontal gyrus right	40.67	1141	6.40	64	24	16
Triangular inferior frontal gyrus right	31.29					
Opercular inferior frontal gyrus right	17.00					
Middle temporal gyrus left	78.63	1095	6.10	− 58	− 32	2
Superior temporal gyrus left	16.71					
Outside	4.66					
Outside	33.33	1338	5.28	22	− 22	4
Putamen right	31.61					
Thalamus right	9.34					
Putamen left	41.29	1194	5.22	− 20	− 12	− 8
Outside	37.02					
Pallidum left	8.04					
Precentral gyrus left	74.31	218	5.10	− 50	− 2	56
Outside	17.43					
Middle frontal gyrus left	7.34					
Supplementary motor area right	37.93	348	4.92	14	− 22	64
Precentral gyrus right	20.98					
Outside	18.97					
Inferior frontal gyrus pars orbitalis right	54.87	339	4.85	38	22	− 10
Insula right	30.97					
Posterior orbital gyrus	8.26					
Outside	53.80	355	4.73	− 12	− 22	60
Paracentral lobule left	20.28					
Precentral gyrus left	14.65					
Supplementary motor area left	82.01	289	4.69	− 8	12	60
Superior frontal gyrus left	7.96					
Supplementary motor area right	6.57					
Inferior frontal gyrus pars orbitalis left	29.97	624	4.48	− 42	22	− 12
Triangular inferior frontal gyrus left	25.64					
Opercular inferior frontal gyrus left	22.92					

Table shows the three regions with the highest number of active voxels within each cluster, labels according to automated anatomic labelling atlas 3 (AAL3), MNI coordinates.

### Exploratory covariate analysis

**Figure 7:**
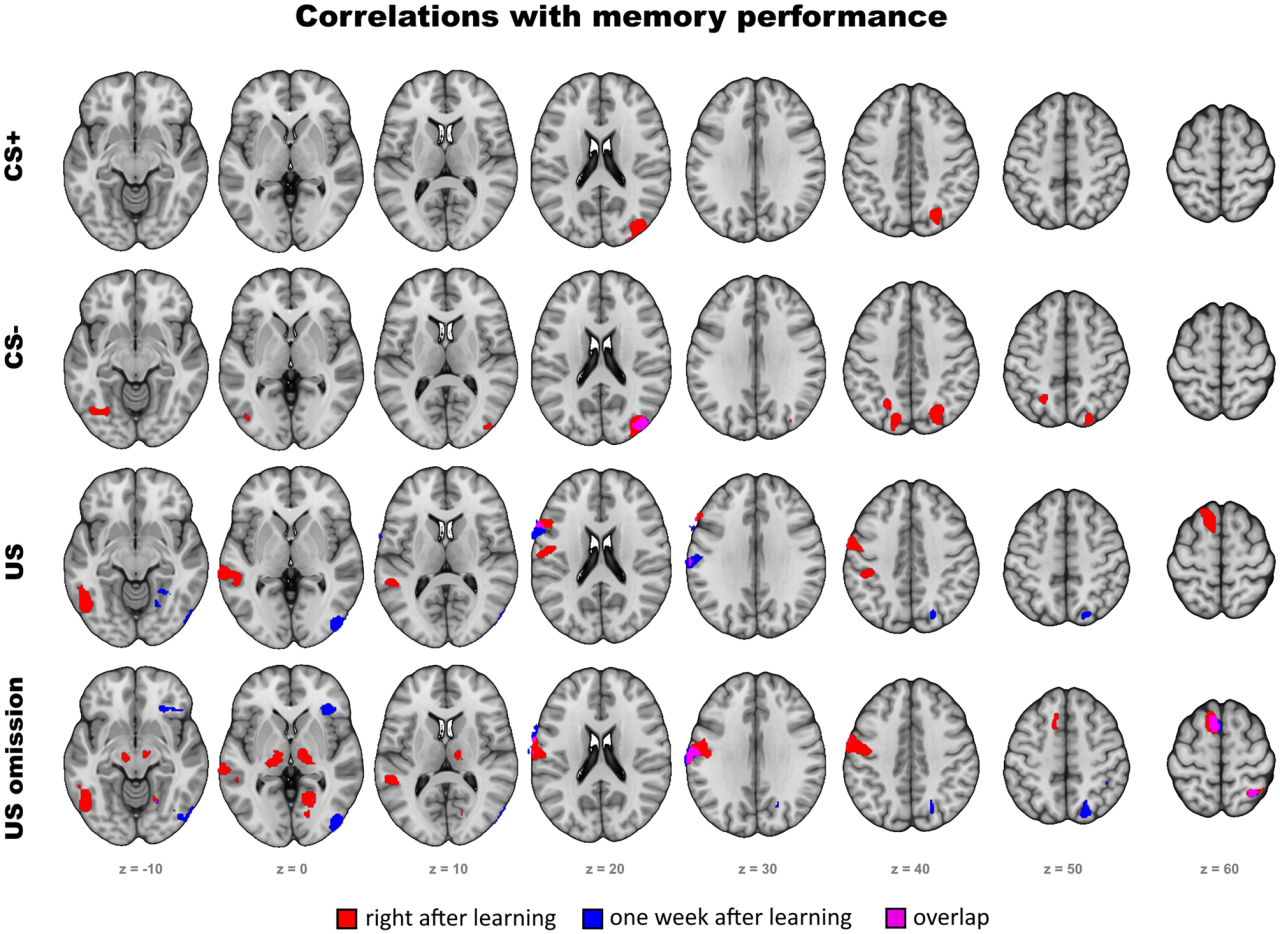
Correlations of brain activity with memory performance. While for CS+ and CS- solely activity in the visual cortex was correlated with memory performance, for US and US omission, also prefrontal and somatosensory areas were correlated with memory performance. Corrected to *p* < .05 by voxel threshold of *p* < .001 and cluster extent of *k* ≥ 126.

In order to analyze how inter-individual differences in brain activity affected subsequent memory of CS-US associations one week after learning, we also conducted an exploratory analysis and introduced memory performance as a covariate on second level. This mainly revealed activity in visual and somatosensory areas in response the US and US omission, both related to performance right after learning and one week later (Fig. 7; Table S1; further details in supplementary material).

## Discussion

In this fMRI study, we examined brain activity related to the explicit associative memory of threat and safety by combining a subsequent memory paradigm with associative threat learning. By separate analyses of neural responses to CS+, CS-, US and US omission, we found subsequent memory effects to facial cues as well as their outcomes. In ROI analyses, we focused on the hippocampus, the vmPFC and the dlPFC, and had a specific interest in the role of US omissions in safety learning. In the following paragraphs, some detailed functional roles of brain areas are discussed based on previous studies, but it is crucial to bear in mind that alternative explanations could be valid and all we can infer from the present results is that these brain areas are related to explicit threat and safety memory.

Consistent with its widely documented role in episodic memory^33,60,61^, the hippocampus was overall associated with subsequent memory, irrespective of the timing of the response or US pairing. Despite of consistent evidence that many memory processes rely on hippocampal activity, its exact functional role is not completely understood yet. It has been proposed that the hippocampus serves as a ‘convergence zone’ in which different neuronal inputs from across the cortex representing different informational fragments come together and preserve a mental image of the environment^61^. These findings of subsequent memory effects in the hippocampus are not self-evident, since previous studies seem to imply that not all memory processes rely on the hippocampus. For example, in case studies, patients with hippocampal lesions showed spared recognition memory, particularly when learning new faces, while the recognition of scenes was impaired^62,63^.

Place cells enable the hippocampus to encode and store complex patterns of spatial configurations^64^. Later, singular traces as parts of a pattern can help to reactivate the whole pattern and thus to remember a scene or the combination of sensory experiences. Transferred to the present experiment, it may be that the hippocampus serves as one ‘convergence zone’ for the visual trace of the CS+ and the somatosensory trace of the US, which enables the re-assignment of a US to a given face, when the face is presented alone. In addition to that, since subsequent memory effects in the hippocampus were not specific to CS+/US associations, it also seems to play a role in the associative memory of a CS- and safety or ‘nothing’.

Related to this, the vmPFC may help generating confident safety memory that is more than the absence of threat memory. We hypothesized that the vmPFC would be associated with subsequent memory and it turned out that vmPFC activity within the ROI was specifically enhanced for remembered CS- and US omissions, but not for CS+ and US responses. The vmPFC is quite consistently activated during the processing of safety signals, such as in the reversal of threat learning^65^, fear extinction^20^, and when a threatening stimulus is at a safe distance^66^. Tashjian and colleagues^67^ propose a Safety Decision Model, in which the vmPFC integrates safety signals by communicating with threat processing areas (insula, amygdala, medial cingulate cortex) and the hippocampus. Neuroanatomically, the vmPFC is strongly connected to the amygdala, setting it in an effective position to down-regulate threat processing^68^. In humans, amygdala-vmPFC connectivity predicts treatment outcome in patients suffering from obsessive–compulsive disorder (OCD)^69^. According to the Safety Decision Model, a self-evaluation process is also incorporated that allows for a safety decision even in the presence of potential threat, if coping abilities are regarded as sufficient to deal with it. It should be noted that what is often referred to as the vmPFC in the literature in fact comprises several subregions of the MPFC which are diverse in cytoarchitecture and possibly regarding functionality^37,67,70^. The current vmPFC ROI was based on meta-analytic findings in the study of fear extinction recall (i.e., enhanced activation for an extinguished vs. unextinguished stimulus)^20^. It can be attributed to the posterior part of the vmPFC or sgACC. It has been suggested that the anterior vmPFC processes safety signals while the posterior vmPFC computes danger signals^67^. Nevertheless, our results strengthen the specific role of this region in safety learning (as opposed to threat learning). After all, the present effect does not reflect safety signaling per se, but subsequent memory of safety associations. However, whole brain analyses further revealed subsequent memory effects in the more anterior part of the vmPFC. Taken together, our results further highlight the role of both the anterior and posterior vmPFC in safety learning, and particularly so in response to the omission of threat and the declarative memory of safety associations. Future studies should try and further elucidate the specific roles of subregions in safety learning.

In the present study, we were specifically interested in the processing of safe outcomes, i.e. US omissions or ‘nothing’ following the CS- and its relationship with subsequent safety memory. Next to the vmPFC, exploratory whole-brain analyses further revealed subsequent memory effects in the more dorsal part of the MPFC, i.e., dmPFC, the occipital gyrus, the fusiform gyrus and the IFG. It is notable that the dmPFC region here should not be mistaken for otherwise observed activity in dorsal anterior cingulate (dACC) which is typically found in threat of shock experiments^71^. Unlike the dACC, the dmPFC here was associated with the absence of threat. However, these findings should be with caution given that this effect was not significantly enhanced in comparison to the subsequent memory effects in the US response. Thus, further studies are needed to test the specificity in the processing of the absence of threat.

Kensinger and colleagues^72^ suggest that the dmPFC contributes to emotional memory by controlling the involved cognitive, social and emotional information, and thereby modulating the affective tone of memory traces. The dmPFC is also involved in executive control processes, such as selecting and updating information, and seems to be particularly active, when socio-emotional content is involved. For example, the dmPFC is more active when the force of a motor action has to be controlled in an emotional vs. a neutral context^73^. Its activity is also enhanced during appraisal strategies in emotion regulation, as a meta-analysis finds^25^, and causally regulates cortical excitability during fear processing^74^. Kensinger and colleagues argue that the connectivity of the dmPFC with other regions involved in emotional processing (amygdala) and semantic memory (temporal lobe) make it well suited for the guidance of emotional memory encoding and retrieval^75^. They also propose an extended model of emotional memory encoding, in which emotions affect memory by modulating memory storage via enhanced amygdala activity and/or by enhanced attention and/or elaboration via the lateral PFC. According to the model, all these processing steps can be influenced by affective appraisals mediated by the dmPFC.

Highly interesting for the present results, the dmPFC plays a role in the stabilization of visual perceptual memory^76^. Along with dmPFC activity, occipital and fusiform gyrus were more active in response to remembered US omissions and the MPFC showed enhanced functional connectivity with visual areas, such as the occipital cortex, the cuneus and the fusiform gyrus. In addition, the paracentral lobule (as the somatosensory representation of the US application to the leg) was also functionally connected to the MPFC. In a previous study, we found that enhanced connectivity between the paracentral lobule and visual cortex in response to the US underlies negative illusory correlations in spider phobia^77^. Due to the exploratory nature of the present connectivity analysis, future studies are warrantable to further examine if and how the conjoint activation of the MPFC and sensory cortices help to correct biased learning processes.

Altogether, in previous studies and proposed models, the dmPFC has been related to (1) maintaining visual perceptual memory, (2) assigning a positive socio-emotional value to memories and (3) facilitating the transfer to semantic memory. Although speculative at this point, similar mechanisms might play a role in establishing declarative safety memory but further studies are needed to confirm these assumptions and to examine their specificity to safety memory.

The third region of interest, the dlPFC, also showed a subsequent memory effect, albeit only the left dlPFC in response to the US and US omission, but not to CS onsets. The dlPFC is closely related to working memory and episodic memory encoding and retrieval^78^. Its function has a causal impact on these processes, as several studies using transcranial magnetic stimulation (TMS) show^79,80^. The present results further add to these findings, that the left dlPFC might be specifically involved in the outcome processing of threat and safety learning. This is interesting from a clinical perspective, because previous research found a connection between the left dlPFC and negatively biased information processing in highly anxious individuals. Balconi and Ferrari^81^ enhanced left dlPFC activity via TMS and found that participants with high trait anxiety showed improved memory of positive stimuli. Similarly, in a previous fMRI study, we found the left dlPFC to be correlated with illusory correlations in spider phobics^44^. The more the dlPFC was enhanced in response to spider pictures, the stronger participants believed that there was a relationship between spider pictures and an aversive US – despite random contingencies. Altogether, the dlPFC might play a role in reducing the biased impact of fear and anxiety on cognition and memory^82^, but it should be kept in mind that the present study only provides correlational evidence.

Next to these regions of interest, on which the focus of this study was lying on, we found additional brain regions to be associated with subsequent threat and safety memory in exploratory whole brain analyses. Most prominently, the IFG was related to memory in both CS+ and CS-, and US omission (also as an inter-individual correlate of memory performance one week after learning). The IFG has been associated with semantic, but also episodic memory retrieval, especially with higher demands on cognitive control^83^. Visual and fusiform cortex (as a central face processing area) might reflect increased attention to and perceptual processing of faces supporting subsequent memory as well^84^. This enhanced activity in visual processing areas is likely also reflected in our previous findings of the late positive potential (LPP) to predict subsequent threat and safety learning^29,30,85^. The basal ganglia have been associated with procedural and stimulus–response learning^86^ and may also play a role in explicit threat learning as the present finding of memory related putamen activity in response to the US suggests.

To summarize our explorative analysis of correlations between brain activity and memory performance across individuals one week after learning, primary visual and somatosensory cortex stood out as covarying regions during learning. While visual cortex activity predicted memory performance in response to CS- and CS outcomes, postcentral gyrus activity predicted memory performance for CS outcomes only. The IFG in response to US omissions was also related to memory performance. Although this analysis differs from our main analysis in terms of sample size and method, it may indicate that lasting threat and safety memory relies on sensory processing and prefrontal control. Further studies will be required to substantiate these findings.

Some clinical implications and hypotheses may be derived from the present results. First, the emergence and maintenance of anxiety disorders may be related to a failure in the discrimination of threat and safety—and particularly to reduced activity of the presently found brain regions such as the MPFC and the hippocampus during the processing of the absence of threat. Indeed, aberrant activity in these regions has been described before in anxiety disorders (e.g.^87,88^). Second, from a treatment perspective, it seems reasonable to test if TMS of the dlPFC can also improve safety memory in anxious individuals. The location of the region is well suited for this method and the present results would predict that TMS should be particularly effective if applied at the moment of US omission. Although, we cannot draw any conclusions about the details of underlying psychological mechanisms in the present study, the recruitment of prefrontal areas would be in line with the idea that the higher order attentional processing of the absence of threat may promote safety learning. This may also be a mediating factor of how the change of threat expectancy during exposure therapy is related to positive treatment outcome^15^. In general, future studies should investigate the effects of cognitive and behavioral interventions at the moment of threat omission, such as appraisal strategies or directed attention.

As a notable limitation of the present experiment, we studied declarative associative threat and safety learning without testing for an actual relationship with fear or anxiety after learning, which might restrict the generalizability of the results to the development of fear and anxiety as opposed to the predominantly cognitive ability to discriminate between threat and safety. We also did not directly assess fear ratings for the CS, so strictly speaking, we know they induced differential arousal and negative affect, but if feelings of fear or anxiety occurred was not controlled for in the current experiment. However, in two prior studies using very similar experimental set-ups, we tested for the re-occurrence of fear in a re-exposure session, which showed that explicit memory was also related to enhanced fear toward the CS+ and reduced fear toward the CS-^29,30^. Finally, the current experimental setting may not be generalizable to some everyday situations, since the explicit intention to learn about threat and safety may not be part of typical adverse experiences. On the other hand, this setting mimics more the situation of the intentional unlearning of threat in the treatment of anxiety disorders, and may thus be more generalizable to this context.

In conclusion, we found that explicit threat and safety learning is related to activity in the hippocampus, the vmPFC and the dlPFC. Building on previous findings, we show that vmPFC activity is specific to safety memory and also involved in US omission response. By focusing on US omission, the present results imply that also the dmPFC might play an important role in safety memory, possibly by stabilizing the integration of cross-sensory stimulation in the visual and somatosensory domain and supporting semantic memory storage. More research is, however, needed to confirm these assumptions. Although this only seems to be a fraction of the underlying processes of threat and safety memory, it helps understanding the neural correlates of remembering the very crucial experience in safety learning—that nothing happened^10^.

### Supplementary Information

Supplementary Information.

## Data Availability

The data analyzed in the current study are available on request from the corresponding author.
