# Supplementary material for: The ventromedial prefrontal cortex in response to threat omission is associated with subsequent explicit safety memory
Source: Sci Rep. 2024 Mar 28;14:7378. doi: 10.1038/s41598-024-57432-0 (PMC10979006; doi:10.1038/s41598-024-57432-0)
Supplement: Supplementary file 1 — Supplementary Information. [file 41598_2024_57432_MOESM1_ESM.pdf]

Supplementary materials for

The ventromedial prefrontal cortex in response to threat omission is associated with subsequent explicit safety memory

Julian Wiemer[1], Franziska Leimeister[1], Matthias Gamer[1] & Paul Pauli[1]

[1] Institute of Psychology (Biological Psychology, Clinical Psychology, and Psychotherapy), University of Würzburg, Würzburg, Germany

Exploratory covariate analysis

In order to analyze how inter-individual differences in brain activity affected subsequent memory of CS-US associations one week after learning, we also introduced memory performance as a covariate in a second level analysis. In particular, we conducted two analyses, one for memory performance immediately after learning and one for memory performance one week after learning. Using such approach, lower trial counts in the remembered category were avoided when memory faded after one week. Eight participants did not return memory ratings after one week, limiting the covariate analyses to 30 participants. We analyzed the identical subsample concerning memory ratings after one week and immediately after learning.

Memory ratings after one week. A repeated measures ANOVA with the factors *CS* (CS+, CS-) and *time* (right after learning, one week after learning) on memory ratings in this subsample revealed a significant main effect of *CS*, $F(1, 29) = 76.01$, $p < .001$, η_p^2=.72, a significant main effect of *time*, $F(1, 29) = 4.42$, $p = .044$, η_p^2=.13, and a significant interaction of *CS* and *time*, $F(1, 29) = 78.27$, $p < .001$, η_p^2=.73. Both right after learning, as well as one week later, CS+ (right after: $M = 1.58 \pm SD = 1.09$; one week later: $M = 0.96 \pm SD = 0.95$) was more associated with the US than the CS- (right after: $M = -1.38 \pm SD = 0.94$; one week later: $M = -0.19 \pm SD = 0.76$), $t(29) = 9.37$, $p < .001$, $d = 1.71$, 95% CI [2.31, 3.61], $t(29) = 6.40$, $p < .001$, $d = 1.17$, 95% CI [0.78, 1.51], respectively. However, the difference between CS+ and CS-, i.e. memory performance, was significantly smaller one week later, $t(29) = 8.83$, $p < .001$, $d = 1.61$, 95% CI [1.40, 2.24].

Correlations with brain activity. Immediately after learning, memory performance was correlated with CS+ related activity in right middle and superior occipital gyrus, fusiform gyrus and left superior occipital gyrus. In a similar vein, CS- related correlation with memory performance was found in right middle and superior occipital gyrus, right fusiform gyrus, left

middle, superior and inferior occipital gyrus and left superior parietal gyrus. Regarding US responses, brain activity was correlated with memory performance in left supplementary motor cortex and superior frontal gyrus, left middle and inferior temporal gyrus, left precentral and postcentral gyrus, left inferior frontal operculum, left inferior parietal, right superior parietal gyrus, right lingual gyrus and the cerebellum. A similar pattern of activations was also found for US omission. Memory performance was correlated in left precentral and postcentral gyrus, left supplementary motor area and middle and superior frontal gyrus, right superior parietal gyrus, left inferior parietal gyrus, left middle and inferior temporal gyrus, left fusiform gyrus, right lingual gyrus, bilateral thalamus, right precentral gyrus and right IFG (see Figure 7 and Table S1).

One week after learning, brain activity displayed a similar picture as immediately after learning, albeit partly fewer regions were significantly activated. For CS+, activity in the right middle occipital gyrus was correlated with memory performance. For the CS-, right middle and superior occipital gyrus and right fusiform gyrus were correlated with memory. In response to the US, brain activity was correlated with memory performance in the right middle and superior occipital gyrus, right fusiform gyrus, left frontal inferior operculum, left inferior temporal gyrus, left middle occipital gyrus, left postcentral gyrus and the cerebellum. In response to US omission, we found activity in the left middle and inferior occipital gyrus, right and left inferior frontal gyrus, left postcentral gyrus, left supplementary motor area and superior frontal gyrus, left inferior temporal and fusiform gyrus, right superior occipital gyrus, right fusiform gyrus, right superior parietal gyrus and right inferior frontal operculum (see Figure 7 and Table S1).

Tables

Table S1. Correlations between brain activity and memory performance.

Region	% of cluster	Cluster size	Peak T-value	Peak voxel x	y	z
**CS+ and memory performance right after learning**						
middle occipital gyrus right	97.89	331	5.24	40	-86	16
superior occipital gyrus right	2.11	331				
superior occipital gyrus right	81.48	189	4.41	24	-70	40
precuneus right	5.82	189				
cuneus right	4.76	189				
**CS+ and memory performance one week after learning**						
no significant cluster						
**CS- and memory performance right after learning**						
middle occipital gyrus right	96.83	473	6.46	42	-84	18
middle temporal gyrus right	1.90	473				
superior occipital gyrus right	0.85	473				
superior occipital gyrus right	64.52	372	5.28	26	-72	40
superior parietal gyrus right	14.78	372				
cuneus right	5.38	372				
superior occipital gyrus left	42.86	147	4.87	-14	-78	44
superior parietal gyrus left	31.29	147				
precuneus left	12.93	147				
superior parietal gyrus left	78.66	164	4.42	-24	-58	44
middle occipital gyrus left	9.76	164				
inferior parietal gyrus left	6.10	164				
fusiform gyrus right	79.37	126	4.37	26	-68	-6
lingual gyrus right	13.49	126				
outside	7.14	126				
inferior occipital gyrus left	46.04	139	4.11	-40	-70	-6
fusiform gyrus left	30.22	139				
middle occipital gyrus left	14.39	139				
**CS- and memory performance one week after learning**						
middle occipital gyrus right	100.00	199	5.16	44	-82	20

US and memory performance right after learning

supplementary motor area left	47.24	381	5.45	-10	22	60
superior frontal gyrus left	43.04	381				
medial superior frontal gyrus left	6.04	381				
inferior temporal gyrus left	69.10	356	4.82	-50	-54	-14
fusiform gyrus left	16.01	356				
inferior occipital gyrus left	13.20	356				
postcentral gyrus left	54.22	166	4.62	-60	-2	40
precentral gyrus left	36.75	166				
outside	9.04	166				
middle temporal gyrus left	94.64	392	4.54	-50	-40	6
superior temporal gyrus left	4.59	392				
outside	0.77	392				
cerebelum	41.09	275	4.22	16	-68	-30
cerebelum crus	27.64	275				
outside	21.45	275				
postcentral left	72.96	196	4.15	-50	-14	24
outside	13.78	196				
rolandic operculum left	8.67	196				
inferior frontal operculum	58.65	208	4.09	-56	24	32
triangular inferior frontal gyrus left	39.42	208				
middle frontal gyrus left	0.96	208				
inferior parietal gyrus left	58.79	165	4.07	-48	-32	34
supramarginal gyrus left	24.24	165				
postcentral gyrus left	11.52	165				

US and memory performance one week after learning

middle occipital gyrus right	46.18	314	6.37	46	-84	0
inferior occipital gyrus right	30.89	314				
middle temporal gyrus right	15.61	314				
opercular inferior frontal gyrus left	52.32	237	4.62	-62	14	26
outside	29.11	237				
precentral gyrus left	8.02	237				
inferior temporal gyrus left	93.21	162	4.43	-50	-50	-16
fusiform gyrus left	6.79	162				
outside	72.52	131	4.36	-30	-74	12
middle occipital gyrus left	27.48	131				
cerebelum right	38.11	475	4.25	20	-62	-20
fusiform gyrus right	33.05	475				
lingual gyrus right	11.16	475				
postcentral gyrus left	73.30	176	4.08	-54	-16	26
supramarginal gyrus left	20.45	176				

outside	6.25	176				
superior occipital gyrus right	44.44	135	4.06	22	-72	44
superior parietal gyrus right	31.11	135				
precuneus right	14.07	135				
**US omission and memory performance right after learning**						
postcentral gyrus left	73.68	1007	5.34	-60	-4	38
precentral gyrus left	16.58	1007				
outside	7.55	1007				
supplementary motor area left	73.75	598	5.25	-8	16	62
superior frontal gyrus left	15.55	598				
medial superior frontal gyrus left	6.69	598				
superior parietal gyrus right	96.14	233	5.16	28	-56	68
inferior parietal gyrus right	3.00	233				
postcentral gyrus right	0.86	233				
middle temporal gyrus left	84.56	298	4.77	-52	-40	8
superior temporal gyrus left	15.44	298				
inferior temporal gyrus left	42.71	679	4.65	-48	-56	-14
fusiform gyrus left	22.09	679				
cerebelum crus left	15.76	679				
lingual gyrus right	34.73	714	4.62	20	-58	-4
cerebelum crus right	23.67	714				
cerebelum right	21.01	714				
outside	22.90	594	4.59	6	-20	-18
ventral lateral thalamus right	17.00	594				
ventral posterolateral thalamus right	15.32	594				
**US omission and memory performance one week after learning**						
inferior occipital gyrus right	22.76	826	7.19	46	-86	2
middle occipital gyrus right	20.34	826				
cerebelum right	17.68	826				
orbital inferior frontal gyrus right	39.63	328	4.80	40	30	-4
insula right	21.65	328				
posterior orbital gyrus right	14.63	328				
postcentral gyrus left	62.63	289	4.77	-66	-18	30
outside	28.37	289				
supramarginal gyrus left	4.84	289				
supplementary motor area left	92.69	342	4.69	-6	14	60
supplementary motor area right	3.51	342				
superior frontal gyrus left	2.34	342				
inferior temporal gyrus left	49.10	334	4.48	-46	-48	-16
fusiform gyrus left	28.14	334				
cerebelum left	15.87	334				
superior parietal gyrus right	88.74	151	4.18	28	-54	68

inferior parietal gyrus right	11.26	151				
superior occipital gyrus right	33.20	244	3.94	22	-72	46
superior parietal gyrus right	29.10	244				
precuneus right	17.21	244				

Table shows the three regions with the highest number of active voxels within each cluster, labels according to automated anatomic labelling atlas 3 (AAL3), MNI coordinates.
